# Early Cardiac Manifestations as the Initial Presentation of Duchenne Muscular Dystrophy in Infancy

**DOI:** 10.7759/cureus.101855

**Published:** 2026-01-19

**Authors:** Feras Shatarat, Jana Alkasasbeh, Rahaf Shatarat, Sofian Tarawneh

**Affiliations:** 1 Medical School, Mutah University, Al-Karak, JOR; 2 Pediatrics, Al-Karak Governmental Hospital, Al-Karak, JOR

**Keywords:** cardiomyopathy, duchenne muscular dystrophy, exon deletion, hyperckemia, left ventricular dilatation

## Abstract

Duchenne muscular dystrophy (DMD) is an X-linked neuromuscular disorder most commonly diagnosed between four and five years of age, while diagnosis during infancy remains uncommon due to initially normal neurological examinations and delayed onset of motor weakness. Although dystrophin deficiency affects cardiac muscle from birth, clinically apparent cardiac involvement is generally considered a later manifestation, rendering echocardiographically evident structural cardiac abnormalities during infancy rare.

We report a male infant with normal early motor development who initially presented at nine months of age with fever, dark urine, elevated transaminases, and markedly increased serum creatine kinase (CK) levels, which were initially attributed to hemolysis and presumed as viral myositis in the context of glucose-6-phosphate dehydrogenase deficiency. CK levels remained persistently elevated, prompting further evaluation. At 12 months of age, neurological examination was normal; however, cardiac assessment revealed mild left ventricular dilatation with preserved systolic function on echocardiography and electrocardiographic features consistent with left ventricular hypertrophy. Genetic testing subsequently confirmed an out-of-frame exon 44 deletion consistent with Duchenne muscular dystrophy. Motor stagnation became apparent by 15 months of age, while serial echocardiographic assessments demonstrated persistent but stable left ventricular dilatation.

This case illustrates an early cardiac presentation of Duchenne muscular dystrophy in which structural cardiac abnormalities preceded overt neuromuscular manifestations. In infants presenting with persistent elevation of serum creatine kinase beyond the expected recovery period of intercurrent illness, further evaluation, including early cardiac assessment, may be clinically important, even when neurological examination and early motor development appear normal.

## Introduction

Duchenne muscular dystrophy (DMD) is the most common and severe X-linked muscular dystrophy, caused by mutations in the DMD gene that result in the complete absence of dystrophin [1-3]. Affected boys typically present in early childhood with delayed motor milestones, progressive proximal muscle weakness, and markedly elevated serum creatine kinase (CK) levels [3-5]. Although dystrophin deficiency affects both skeletal and cardiac muscle from birth, clinically apparent cardiomyopathy is generally regarded as a later manifestation, most commonly emerging during school age or adolescence in the form of dilated cardiomyopathy, ventricular enlargement, and impaired systolic function [1,3,6-8].

Importantly, myocardial involvement begins well before overt cardiac symptoms become evident. Advanced imaging studies have demonstrated early structural and functional myocardial alterations preceding clinical cardiomyopathy, reflecting subclinical myocardial injury in patients with DMD [6,7,9,10]. Despite this early pathophysiological involvement, clinically recognizable cardiac abnormalities during infancy remain uncommon and are rarely the initial manifestation of the disease [8,11,12].

Early identification of cardiac involvement has become a central focus of contemporary DMD care guidelines, supported by evidence that timely cardioprotective therapy may delay the progression of DMD-associated cardiomyopathy [7,8,10]. However, diagnosis during infancy is frequently delayed, as elevated muscle enzymes are often misattributed to benign or transient conditions such as viral myositis or intercurrent illness. In infancy, elevations in serum creatine kinase (CK) are frequently misattributed to viral myositis or intercurrent illness, as aspartate aminotransferase (AST) and alanine aminotransferase (ALT) may rise in muscle injury, creating a diagnostic pitfall that often delays recognition of dystrophinopathy [4,13]. 

Current consensus recommendations generally advise routine cardiac surveillance in Duchenne muscular dystrophy beginning in early childhood, using echocardiography or cardiac magnetic resonance imaging to detect subclinical myocardial involvement [14]. In this context, the identification of echocardiographically evident structural cardiac abnormalities during infancy represents an uncommon finding and underscores the novelty of the present case.

Here, we report a case in which early structural left ventricular abnormalities, rather than neuromuscular weakness, represented the first clinical clue to Duchenne muscular dystrophy at 12 months of age. This case underscores the diagnostic value of cardiac evaluation in infants with persistent elevation of serum creatine kinase and highlights the importance of considering dystrophinopathy even in the absence of overt neurological deficits or abnormal early motor development.

## Case presentation

Patient information

A male infant was born at term via spontaneous vaginal delivery following a pregnancy complicated by maternal COVID-19 infection and intrauterine loss of a co-twin. The neonatal course was largely unremarkable, aside from transient neonatal jaundice requiring phototherapy. Early development during infancy was age-appropriate, including cruising and supported ambulation by 12 months without independent walking. The patient had a known diagnosis of glucose-6-phosphate dehydrogenase (G6PD) deficiency and no family history of neuromuscular or inherited cardiac disease.

Clinical findings and presentation

At nine months of age, he presented with fever and dark urine in the context of known G6PD deficiency, raising concern for hemolysis. Laboratory evaluation demonstrated biochemical features compatible with hemolysis but also unexpectedly revealed markedly elevated muscle enzymes, including a serum CK level of 5,211 U/L, elevated lactate dehydrogenase (LDH), and an aspartate aminotransferase (AST) level higher than alanine aminotransferase (ALT) (AST 225 U/L > ALT 174 U/L), as summarized in Table 1. This constellation of markedly elevated serum CK, AST-predominant transaminase elevation, and elevated LDH was interpreted as consistent with presumed viral myositis in the setting of an intercurrent febrile illness. Given the coexistence of known G6PD deficiency, an episode of dark urine, a normal neurological examination, and the absence of a family history of neuromuscular disease, this explanation was considered the most clinically plausible at the time, and dystrophinopathy was not initially suspected. Follow-up serum creatine kinase testing obtained approximately two months later demonstrated persistently elevated levels rather than the expected normalization. At 11 months of age, the persistence of CK elevation raised concern for an underlying myopathic process. Because sustained elevation of serum creatine kinase is inconsistent with uncomplicated viral myositis, this finding prompted referral for further neurological and cardiac evaluation.

**Table 1:** Initial laboratory findings at nine months of age CK = creatine kinase; AST = aspartate aminotransferase; ALT = alanine aminotransferase; LDH = lactate dehydrogenase; WBC = white blood cell count

Test	Result	Reference Range	Comment
CK	5,211 U/L	30–200 U/L	Severely elevated
AST	225 U/L	20–67 U/L	Markedly elevated
ALT	174 U/L	8–45 U/L	Markedly elevated
LDH	1185 U/L	120-246 U/L	Markedly elevated
Sodium	136 mmol/L	139–146 mmol/L	Slightly low
WBC	16.7 ×10³/µL	6–13.5 ×10³/µL	leukocytosis
Hemoglobin	12.5 g/dL	10.1–12.5 g/dL	Normal
Platelets	390 ×10³/µL	206–445 ×10³/µL	Normal
Bilirubin (total)	10.2 µmol/L	0–17.1 µmol/L	Normal
Albumin	4.6 g/dL	2.8–4.7 g/dL	Normal
Hepatitis panel	Negative	—	Excludes viral hepatitis

Diagnostic assessment

At 12 months of age, neurological assessment documented a completely normal neuromuscular examination, including normal muscle tone, preserved deep tendon reflexes, intact cranial nerve function, and no clinical evidence of weakness or motor regression.

**Figure 1:**
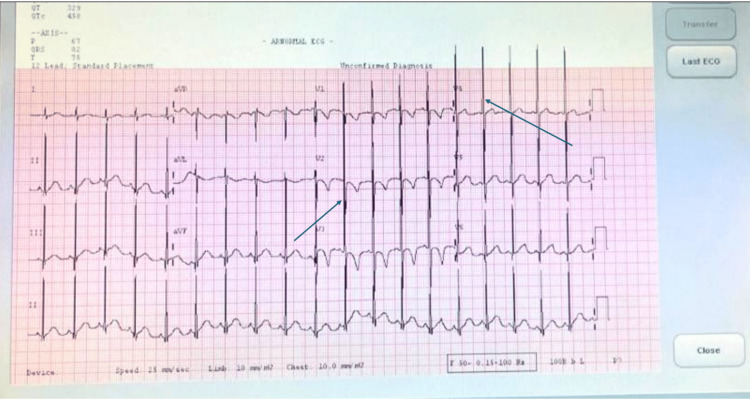
Electrocardiogram demonstrating left ventricular hypertrophy in an infant with Duchenne muscular dystrophy Twelve-lead electrocardiogram obtained at 12 months of age demonstrating sinus rhythm with voltage criteria consistent with left ventricular hypertrophy. Arrows highlight deep S waves in the right precordial leads and tall R waves in the left precordial leads, consistent with early structural cardiac involvement in Duchenne muscular dystrophy.

Cardiac evaluation represented the first objective abnormality. Transthoracic echocardiography demonstrated mild left ventricular dilatation, with a left ventricular end-diastolic diameter (LVEDD) of 33.5 mm (Z-score +2.0) and a left ventricular end-systolic diameter (LVESD) of 23 mm (Z-score +3.2). Left ventricular systolic function was preserved, with an ejection fraction of 59%. Electrocardiography demonstrated voltage criteria consistent with left ventricular hypertrophy (Figure 1).

Taken together, these findings were suggestive of early structural cardiac involvement with preserved systolic function in an otherwise asymptomatic infant.

Given the coexistence of persistently elevated serum CK levels and early left ventricular remodeling in the absence of neurological deficits, the findings were reviewed in a multidisciplinary clinical context. Although formal heart failure staging systems are not routinely applied in infancy, the presence of structural cardiac abnormalities with preserved systolic function was considered indicative of a preclinical cardiac phenotype. This prompted further etiologic evaluation for an underlying neuromuscular disorder, including targeted genetic testing for dystrophinopathy.

Multiplex ligation-dependent probe amplification (MLPA) performed at 13 months of age identified a hemizygous out-of-frame deletion of exon 44 in the dystrophin (DMD) gene, confirming the diagnosis of Duchenne muscular dystrophy.

Therapeutic intervention

Despite the presence of mild left ventricular dilatation, cardioprotective pharmacotherapy was not initiated, as the patient remained asymptomatic with preserved systolic function and stable ventricular dimensions. This management approach was consistent with current pediatric DMD cardiac surveillance recommendations, which typically reserve initiation of angiotensin-converting enzyme inhibitors, beta-blockers, or mineralocorticoid receptor antagonists for patients with progressive ventricular dilatation, systolic dysfunction, or cardiac magnetic resonance evidence of myocardial fibrosis.

At 18 months of age, oral prednisolone therapy was initiated as part of standard Duchenne muscular dystrophy management, with good tolerance. The patient was enrolled in a structured care program including regular neurology and cardiology follow-up, physiotherapy, occupational therapy, and aquatic therapy. The family also received counseling regarding potential eligibility for future exon-skipping gene-targeted therapies.

Follow-up and outcomes

Although neurologically stable at diagnosis, follow-up at approximately 15 months revealed motor stagnation, with no progression beyond supported standing and assisted ambulation. Independent walking was achieved at approximately 20 months but was accompanied by frequent falls and early loss of endurance, marking the onset of overt neuromuscular involvement and the first clear clinical evidence of neuromuscular progression. Cardiac reassessment at the same visit demonstrated persistent but unchanged mild left ventricular dilatation with preserved systolic function, consistent with stable early structural cardiac involvement.

A subsequent echocardiogram at 22 months confirmed ongoing mild left ventricular dilatation (LVEDD 35.3 mm, Z-score +2.0; LVESD 22.7 mm, Z-score +2.04) with preserved ejection fraction (67%), trivial tricuspid and pulmonary regurgitation, normal septal anatomy, normal atrioventricular and ventriculo-arterial connections, and no pericardial effusion.

At the most recent follow-up at 32 months of age, the patient demonstrated progressive neuromuscular involvement, including increased proximal muscle weakness, early swallowing difficulty with occasional choking on solids, global developmental delay, and significant expressive speech delay limited to two to three single words. Despite neuromuscular progression, cardiac function remained stable under continued surveillance, with no clinical signs of heart failure.

## Discussion

Early diagnosis of Duchenne muscular dystrophy in infancy

Early diagnosis of DMD during infancy remains uncommon, as overt neuromuscular weakness typically becomes clinically apparent only after significant skeletal muscle degeneration has occurred. Most patients are diagnosed between four and five years of age, and diagnoses before two years account for only a small minority of reported cases, usually reported in isolated case series or individual reports [3,5,11]. When DMD is identified in infancy, it is most often triggered by incidental laboratory abnormalities, such as persistent elevation of serum creatine kinase or unexplained elevation of aminotransferases, rather than by motor delay or weakness [4,5].

In contrast to the typical diagnostic pathway, our patient demonstrated entirely normal early motor development and a normal neurological examination at the time of diagnosis, highlighting the diagnostic difficulty of DMD in very young infants without clinical weakness or a contributory family history.

Although newborn screening for Duchenne muscular dystrophy is not universally implemented, this case highlights the relevance of incidental biochemical findings, such as persistent hyperCKemia, as a potential pathway to earlier diagnosis in infancy.

Diagnostic pitfalls: misattribution of elevated creatine kinase to benign conditions

Elevation of serum creatine kinase in infancy is frequently misattributed to transient or benign causes, including viral myositis, post-infectious muscle injury, and dehydration. In the present case, the coexistence of fever, dark urine, known G6PD deficiency, and an AST-predominant transaminase pattern created a clinically plausible explanation for viral myositis and hemolysis, delaying consideration of an inherited myopathy. Similar diagnostic delays have been described in prior reports, emphasizing that persistence of elevated creatine kinase beyond the expected recovery period of viral myositis, typically several weeks, should raise concern for an underlying myopathic process. In infancy, longitudinal trends in creatine kinase levels are often more informative than isolated absolute values [4,5].

Early cardiac involvement and cardiac-first presentation

Cardiac involvement in Duchenne muscular dystrophy has traditionally been considered a later manifestation, typically emerging during school age or adolescence as dilated cardiomyopathy or myocardial fibrosis. However, accumulating evidence indicates that dystrophin deficiency leads to early cardiomyocyte fragility, calcium dysregulation, and microvascular injury long before overt systolic dysfunction becomes clinically apparent [6-8].

Although subclinical myocardial changes are known to begin early, clinically detectable structural cardiac abnormalities during infancy remain exceptionally rare. Cardiac-first presentations, in which cardiac abnormalities precede overt neuromuscular manifestations, have been reported only sporadically and almost exclusively outside infancy [4,8]. In this context, the findings observed in our patient are consistent with early structural cardiac involvement accompanied by preserved systolic function, representing a preclinical cardiac phenotype preceding overt cardiomyopathy.

Unlike previously reported early or subclinical cardiac changes, which are often detectable only by advanced imaging or precede overt structural remodeling, the echocardiographic finding of left ventricular dilatation in infancy represents a clinically apparent structural abnormality. While left ventricular dilatation in the absence of myocardial fibrosis or systolic dysfunction does not necessarily imply immediate adverse prognosis, it may reflect early remodeling that warrants close longitudinal surveillance.

Multidisciplinary evaluation as a diagnostic catalyst

A defining aspect of this case was the pivotal role of multidisciplinary evaluation in establishing the diagnosis. Despite a completely normal neurological examination, cardiology assessment revealed mild left ventricular dilatation with preserved systolic function, along with electrocardiographic evidence of left ventricular hypertrophy. These cardiac abnormalities raised suspicion for an underlying neuromuscular etiology and directly prompted targeted genetic testing, ultimately enabling a definitive diagnosis. This diagnostic sequence underscores the importance of incorporating cardiac assessment into the evaluation of infants with persistent elevation of serum creatine kinase, even in the absence of overt neurological deficits or clinical weakness [6,8].

In this case, cardiology involvement was guided by individual clinical judgment rather than a predefined protocol; however, this diagnostic sequence may inform future multidisciplinary approaches for infants with persistent hyperCKemia.

Clinical course and implications for early surveillance

The subsequent emergence of motor stagnation at approximately 15 months confirmed progression into the early symptomatic neuromuscular phase, validating the clinical significance of the early cardiac findings. Longitudinal follow-up demonstrated stable early structural cardiac involvement with preserved systolic function, consistent with observations that cardiac remodeling may precede functional decline by several years [6,7].

Early identification of DMD provides a critical window for structured surveillance, multidisciplinary care, and timely initiation of standard therapies, including corticosteroids and anticipatory cardiac monitoring. While cardioprotective pharmacotherapy was not initiated in this case due to preserved systolic function, early recognition allowed for close follow-up in accordance with established cardiac management frameworks [6,7].

In this case, cardiac surveillance intervals were aligned with contemporary pediatric DMD cardiac monitoring recommendations. Although cardioprotective therapy was deferred due to preserved systolic function, early structural findings may influence the timing of future therapy initiation as part of individualized longitudinal management.

## Conclusions

This case highlights an important clinical lesson in the evaluation of infants with persistent hyperCKemia or unexplained aminotransferase elevation. Duchenne muscular dystrophy should be considered in the differential diagnosis even in the absence of overt neuromuscular weakness or abnormal early motor development. Incorporating cardiac assessment into the diagnostic workup may uncover early structural abnormalities that provide a critical diagnostic clue to an underlying dystrophinopathy and facilitate timely genetic confirmation. Early structural cardiac changes may represent the earliest clinical manifestation of Duchenne muscular dystrophy in infancy. Recognition of cardiac-first presentations underscores the importance of multidisciplinary collaboration among pediatricians, cardiologists, and neurologists to avoid diagnostic delay.

This case broadens current understanding of early DMD phenotypes and supports proactive cardiac evaluation as part of the assessment strategy for infants with persistent muscle enzyme abnormalities. From a practical standpoint, this case suggests that consideration of cardiac evaluation may be warranted in infants with persistent hyperCKemia or unexplained AST elevation, even in the absence of overt neuromuscular weakness. Rather than supporting a change in existing diagnostic algorithms, this observation primarily serves to raise clinical awareness of cardiac-first presentations of Duchenne muscular dystrophy in infancy.
